# Cannabidiol’s cytotoxicity in pancreatic cancer is induced via an upregulation of ceramide synthase 1 and ER stress

**DOI:** 10.1186/s42238-024-00227-x

**Published:** 2024-05-08

**Authors:** Nagina Mangal, Vikash Reebye, Nagy Habib, Mikael H. Sodergren

**Affiliations:** 1 Medical Cannabis Research Group, Department of Surgery and Cancer, Imperial College London, London, W12 0NN UK; 2 Systems and Precision Cancer Medicine Team, Division of Molecular Pathology, Institute of Cancer Research, Sutton, SM2 5NG UK

**Keywords:** CBD, Cannabidiol, Pancreatic cancer, PDAC, Ceramide, CerS1, Ceramide synthase 1, Endoplasmic reticulum stress

## Abstract

Pancreatic ductal adenocarcinoma (PDAC) remains one of the most aggressive malignancies with a median 5 year-survival rate of 12%. Cannabidiol (CBD) has been found to exhibit antineoplastic potential and may potentiate the anticancer effects of cytotoxic’s such as gemcitabine. CBD therapy has been linked to *de novo* synthesis of ceramide. The sphingolipid ceramide is a potent tumour suppressor lipid with roles in apoptosis and autophagy. One of the key players involved is ceramide synthase, an enzyme with six isoforms (CerS1-CerS6), reported to have disease prognostic value. Quantitative real time PCR was used to determine mRNA expression levels of ceramide synthase isoforms, GRP78, ATF4 and CHOP. Western blotting was used to analyze protein expression of these markers and knockdown of CerS1 and GRP78 were applied via an siRNA and confirmed by the two mentioned methods. Mice with PDAC xenografts were injected via intraperitoneal method with drugs and tumours were analysed with flow cytometry and processed using H&E and IHC staining. siRNA knockdown of ceramide synthase 1 (CerS1) and analysis point to evidence of a putative CerS1 dependent pathway driven by CBD in activating endoplasmic reticulum (ER) stress target; GRP78. Upon CBD treatment, CerS1 was upregulated and downstream this led to the GRP78/ATF4/CHOP arm of the unfolded protein response (UPR) pathway being activated. In an in vivo model of PDAC in which CerS1 was not upregulated on IHC, there was no observed improvement in survival of animals, however a reduction in tumour growth was observed in combination chemotherapy and CBD group, indicating further investigations *in vivo.* These findings provide evidence of a potential ceramide induced cytotoxic mechanism of action of CBD in pancreatic ductal adenocarcinoma.

## Introduction

Ceramide synthase 1 (CerS1), however pancreatic ductal adenocarcinoma (PDAC) remains as one of the most aggressive malignancies with a median 5 year-survival rate of 12% (Puckett and Garfield, 2022). Until now chemotherapy remains as the major source of treatment for locally advanced and metastatic stages however this has not significantly improved survival outcomes. Plant-derived cannabinoids which include Cannabidiol (CBD) have emerged as possible bioactive molecules possessing anti-tumour properties (Perez-Mancara et al., 2012). Cannabinoids major first step in their downstream antitumour effects is through upregulating the *de novo* synthesis pathway of ceramide generation (Gómez del Pulgar et al., 2002; Mangal et al. 2021; Sun et al. 2023). The sphingolipid ceramide is a potent tumour suppressor lipid with roles in proliferation, apoptosis, migration, and autophagy. These lipids consist of a long chain sphingosine backbone amide-linked to a fatty acyl chain which varies in length, and this is determined by specific ceramide synthases (CerS) of which 6 exist (Ceramide synthase 1–6; CerS1-6) and determines the specific functions of the sphingolipid in the cell (Raichur 2020). Ceramide which is the key sphingolipid regulates selective autophagy of the mitochondria (mitophagy). Aberrations to mitophagy have implications on cancer cell proliferation, chemotherapy response and cell death (Raichur 2020).

For the synthesis and correct folding of membrane and secreted proteins to happen, the *de novo* pathway is initiated, and this takes place in the endoplasmic reticulum (ER) (Piña et al. 2018). However, this process can go awry if the build-up of misfolded proteins is greater in the ER which causes an imbalance of these misfolded proteins leading to an overall dysfunction to critical cellular functions observed in cancer (Chen and Cubillos-Ruiz 2021). ER stress can trigger the unfolded protein response (UPR) which is a quality control system essential to ER homeostasis (Park and Park 2020; Chen and Cubillos-Ruiz 2021). The UPR is able to adapt to high stress situations, however if the misfolded proteins become impossible to correct then a process known as ER-associated protein degradation (ERAD) is executed (Hwang and Qi 2018). The UPR is controlled by the three ER-transmembrane stress sensors: inositol-requiring enzyme 1α (IRE1α), pancreatic endoplasmic reticulum kinase (PERK), and activating transcription factor 6 (ATF6). PERK and IRE1α pathway, although mainly IRE1α, leads to apoptosis if there is prolonged ER stress, through a proposed mechanism through transcription factor E2F1. Both the downregulation of E2F, a transcription factor (E2F1) and increased CAAT/enhancer-binding protein (CHOP) a proapoptotic protein, expression by ATF6α bring the cell to a point of no return and induce apoptosis.

In cancer, UPR is a key factor in the context of signalling pathways controlling the progression, metastasis, and survival of the tumour (Park and Park 2020; Chen and Cubillos-Ruiz 2021). UPR becomes activated in response to the highly proliferative metabolic rate of the cancer cells which enhances misfolding of ER proteins (Hwang and Qi 2018). The UPR can therefore function as a pro-survival route for tumour cells in adopting the adaptive mechanisms for tumour progression (Hetz and Papa 2018). Remarkably, the time-based property of UPR makes it an attractive target for anticancer therapy in that a short period resolves ER homeostasis but a persistent one leads to apoptosis (Hetz and Papa 2018).

Interestingly, sphingolipids which are at the centre of ER stress can determine the type of ER stress response due to the length of its acyl chain (Piña et al. 2018; Ho et al. 2022). For example, CerS1 and CerS6 which generate C18- and C16-ceramides respectively were shown to have both pro-apoptotic and pro-survival roles in head and neck squamous cell carcinoma (HNSCC) (Senkal et al. 2007, 2010). Their findings show the activation of the ER stress-mediated apoptosis via ATF6/CHOP branch of the UPR pathway when CerS6 was knocked down via siRNA (Senkal et al. 2007, 2010). However, in their in vivo model, CerS6/C16-ceramide protected ER stress and tumour growth was enhanced in comparison to CerS1/C18-ceramide (Senkal et al. 2007, 2010). These findings highlight the different roles acyl chain lengths of CerS in ER mediated stress can play and additionally studies have reported specific effects of CerS and its derivatives in ER stress may depend on the cell and cancer type.

In vivo reports of CBD’s antitumour effects have been evident when administered in combination with other therapies such as chemo toxic agents, radiotherapy and or in combination of all. An in vivo study carried out by Ferro et al., showed in a mouse model of PDAC, that animals treated with a combination of CBD and gemcitabine prolonged their survival by three times more in comparison to gemcitabine single treatment (Ferro et al. 2018).

CBD has been reported previously to induce ER stress in many cancer models through various stress inducing factors such as hypoxia, starvation, pH changes (Solinas et al. 2013; Velasco et al. 2016). Moreover, evidence has shown cannabinoids favouring ER stress through a ceramide inducing mechanism (Gómez del Pulgar et al., 2002; Park and Park 2020). Additionally, the upregulation of ER stress through ceramide biosynthesis via cannabinoid-based formulations have been reported to overcome the chemoresistance in cancer (Go et al. 2020). Therefore, combining chemotherapeutic drugs with cannabinoids may help to reverse this chemoresistance through targeting this mechanism. Based on the evidence, in this study we evaluated the expression levels of ceramide synthase isoforms 1–6, which revealed CerS1 as a significant upregulated target following CBD treatment as a monotherapy and in combination with gemcitabine. In order to elucidate a cytotoxic mechanism of action, knockdown of CerS1 revealed GRP78, ATF4 and CHOP as downstream apoptotic initiating signals.

## Results

### Cannabidiol reduces pancreatic cancer growth in a dose and time dependent manner

To determine whether CBD could affect cell viability and hence induce cytotoxicity in pancreatic cancer cell lines, a variety of pancreatic cancer cell lines were treated with cannabidiol and gemcitabine over timepoints ranging from 24 to 72 h. Cell viability assay showed cannabidiol could reduce cell viability in a time and dose-dependent manner as shown in Table 1.

**Table 1:** IC_50_values of screened cell lines

Cell line	48 h	72 h
h- Panc03.27	CBD – 32.32 ± 0.3	CBD – 26.51 ± 0.9
	GEM – 0.012 ± 0.2	GEM – 0.008 ± 0.5
h- Panc1	CBD – 20.8 ± 0.9	CBD – 19.9 ± 0.6
	GEM – 0.53 ± 0.7	GEM – 0.13 ± 0.7
m-3275	CBD – 21.4 ± 0.5	CBD – 17.19 ± 0.8
	GEM – 0.06 ± 0.3	GEM – 0.04 ± 0.5
h- HPAF-II	CBD – 23.4 ± 0.9	CBD – 18.8 ± 0.1
	GEM – 0.25 ± 0.4	GEM – 0.09 ± 0.4
h- CFPAC-I	CBD – 14.9 ± 0.1	CBD – 12.7 ± 0.9
	GEM – 0.013 ± 0.3	GEM – 0.009 ± 0.6
h-H6c7 (HPDE)	CBD – 55 ± 0.2	CBD – 51 ± 1.2
h-IMR-90	CBD – 50 ± 0.7	CBD – 47 ± 0.4

Various pancreatic cancer cell lines both human (h-) and murine (m-) and IMR-90 inhibitory concentration values following treatment with CBD and GEM = gemcitabine, over 48 and 72 h. Data shown as µM and represents three independent experiments performed as quadruplicates as IC_50_ ± SEM values from cell-titre glo assay (SRB assay produced similar IC_50_, data not shown)

**Fig. 1:**
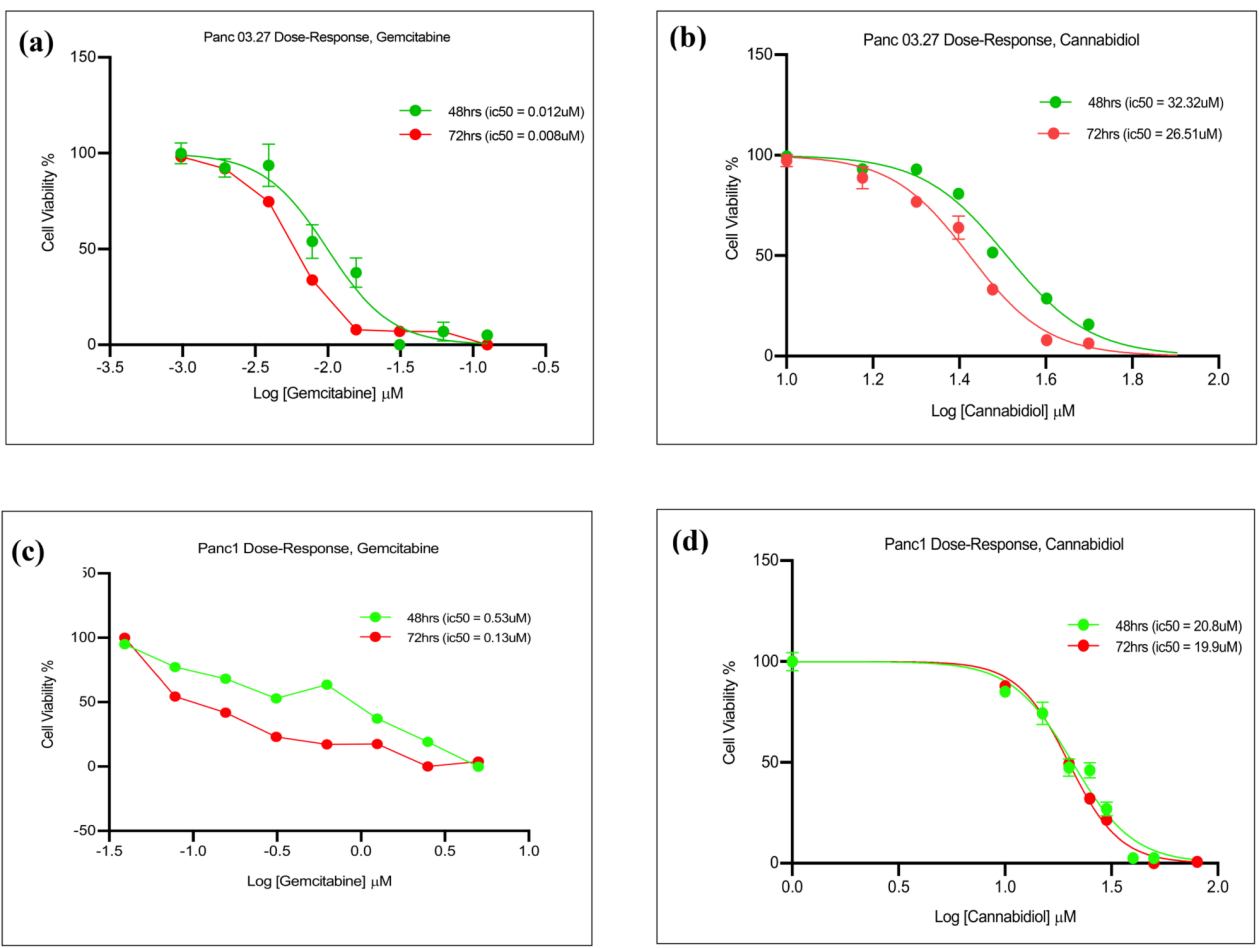
Cannabidiol (CBD) reduces cell viability in pancreatic cancer cells. (**a**) Dose response curves representing 48 and 72 h treatment of drugs gemcitabine (**a**), (**c**) and CBD (**b**), (**d**) in human Panc03.27 and Panc1 cells respectively

Panc03.27 and Panc1 were further explored to evaluate whether cell lines with varying gemcitabine sensitivity showed differences to CBD treatment (see Fig. 1).

### CerS1 is upregulated by CBD in its cytotoxic mechanism of action

**Fig. 2:**
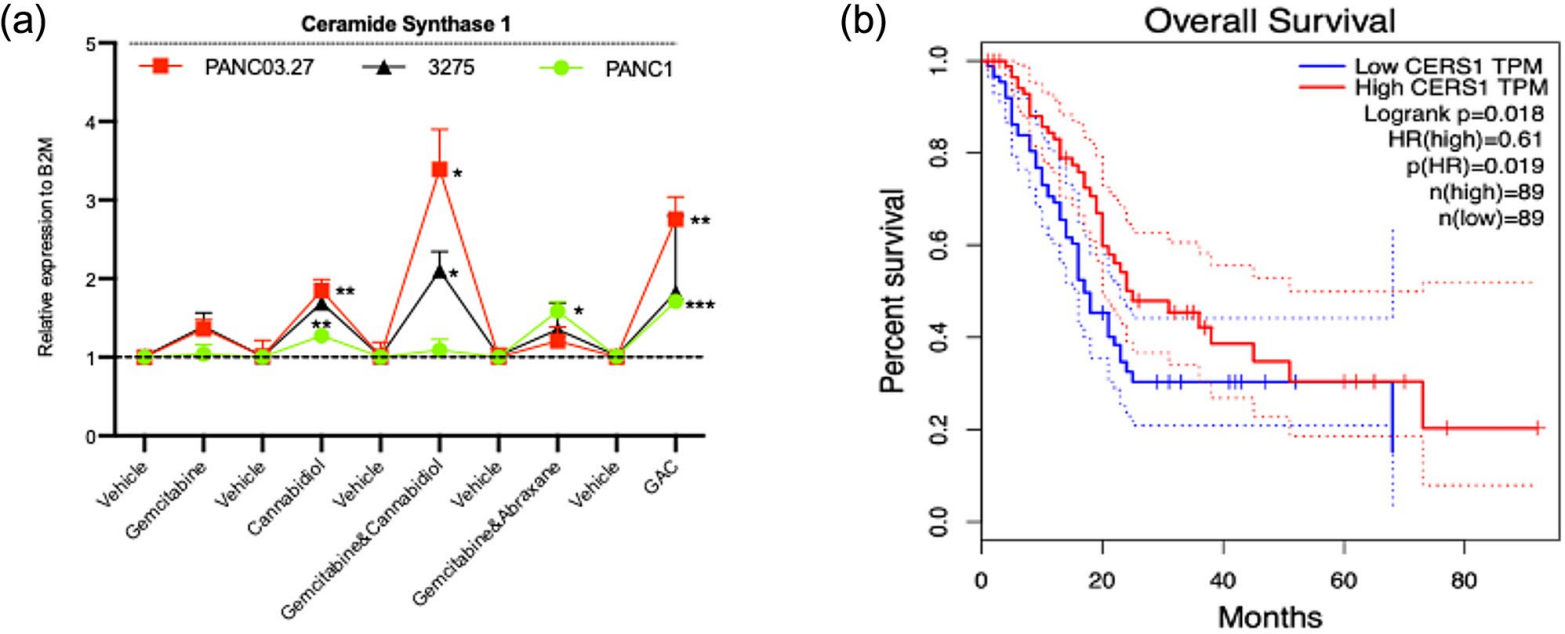
Ceramide Synthase isoform 1 is upregulated by cannabidiol in pancreatic cancer. (**a**) Summary plot of mean and error with SD of CerS1 fold changes across all treatment groups of concentraions reflected in table 1’s ic50 values at 48 h, comparing human Panc03.27, Panc1 and murine-3275 cells. The subsequent statements indicate greatest to lowest significance of CerS1 upregulation with gemcitabine, abraxane and CBD (GAC) treatment in Panc03.27 cells followed by CBD treatment, then combination of gemcitabine and CBD (GC) and finally gemcitabine single treatment. In Panc1 cells, GAC treatment followed CBD single treatment and finally gemcitabine and abraxane (GA). Murine 3275 cells showed signficance in CerS1 upregulation in GC combination treatment only in comparison to control. (**b**) Using publicly available RNA sequencing data (GSEA, gene set enrichment analysis) of PDAC tumours versus normal, a Kaplan-Meier curve indicates higher CERS1 correlates with better overall survival (OS) (HR: hazard ratio = 0.61, Logrank *p* = 0.018, *p* = 0.019). Gem = gemcitabine, Abx = abraxane, CBD = cannabidiol

As previously discussed, CBD has been reported to cause an upregulation of ceramide via the *de novo* synthesis pathway. Investigation into a sub-class of enzymes ceramide synthases were evaluated in the cell lines of interest. The results showed that in human Panc03.27 and Panc1 cells and murine cell line (3275), ceramide synthase 3 (CerS3) isoform was not detected on qPCR using both Qiagen and TaqMan probes. CerS1, however, was significantly upregulated across all cell lines where various drug treatment groups also exhibited differences in mRNA expression of CerS1 (Fig. 2a). In gemcitabine sensitive cells, Panc03.27, gemcitabine and CBD alone exhibited a 1.36 and 1.85-fold upregulation respectively, triple therapy group which included gemcitabine, abraxane and CBD displayed a 2.27-fold upregulation and combination of CBD and gemcitabine showed a significant 3.39-fold upregulation.

In gemcitabine resistant cells, Panc1, gemcitabine and CBD single treatments showed a 1.04 and 1.27-fold upregulation in mRNA expression of CerS1 respectively. CBD and gemcitabine combination exhibited a 1.59-fold upregulation followed triple combination group of gemcitabine, abraxane and CBD which exhibited a significant 1.71-fold upregulation. In contrast, murine pancreatic cancer cell line (3275) revealed highest mRNA expression of CerS1 in CBD treatment group (2.44-fold upregulation), followed by combination of gemcitabine and CBD (2.10-fold upregulation), triple combination group (1.83-fold upregulation) and gemcitabine monotherapy showed a 1.39-fold upregulation (Fig. 2a). Considering these findings, investigation of CerS1 expression levels in other cancer models have shown both high and low levels associated with poor prognosis. However, in pancreatic cancer, using publicly available RNA sequencing expression data (Tang Z et al., 2017), revealed that a higher expression of CerS1 significantly correlated to better overall survival (OS) (Fig. 2b).

### Mechanism of action of CBD differs across pancreatic cancer cell models

**Fig. 3:**
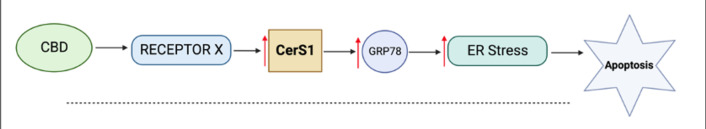
Schematic diagram showing the mechanism of CBD in inducing CerS1 upstream of UPR and cell death. CBD acts through a receptor, currently unknown and enhances the upregulation of CerS1 and this initiates ER stress through master regulator GRP78 which activates UPR signalling, and this leads to downstream cell death

In order to validate the observed CerS1 upregulation and determine cytotoxic mechanism action of CBD, an siRNA was used to knockdown CerS1 and downstream targets of ER stress; GRP78, CHOP and ATF4 were analysed. The results show an overall mechanism of CBD’s cytotoxic effects through upregulation of CerS1, activation of GRP78, ATF4 arm of the UPR pathway further resulting in elevated CHOP expression which induces ER stress leading to apoptosis through a possible cannabinoid receptor which remains unknown (Fig. 3).

#### Knockdown of CerS1 does not correlate with ER stress in Panc03.27 cells

**Fig. 4:**
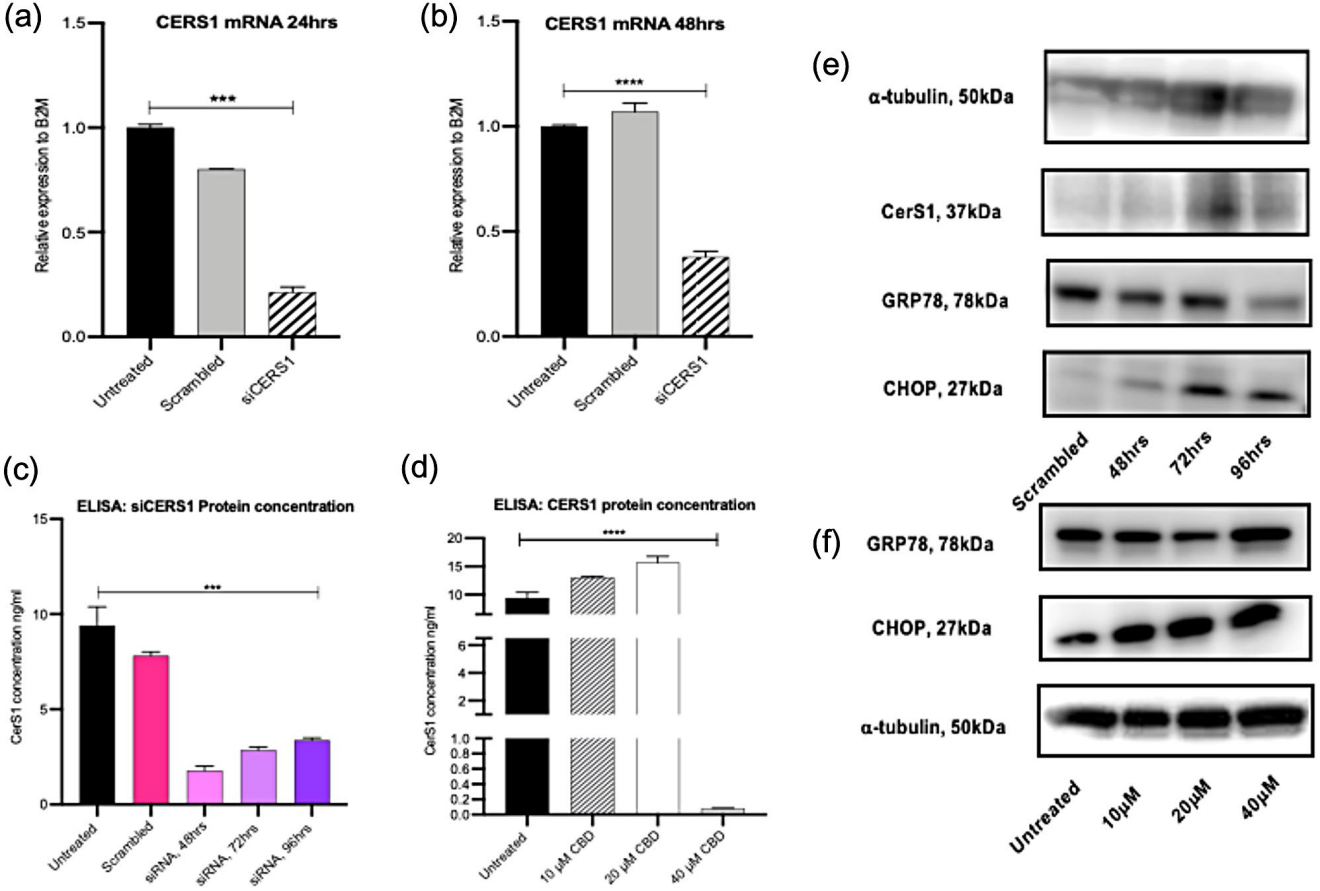
Ceramide synthase 1 knockdown reduces protein expression of GRP78 in Panc03.27 cells. (**a**) and (**b**) qPCR for CerS1 mRNA following transfection with 5 nM si-CERS1 for 24 and 48 h. (**c**) Bar blot of protein concentration (ng/ml) of CerS1 using ELISA following transfection with 5 nM si-CERS1 for 48, 72 and 96 h. (**d**) Bar blot of protein concentration (ng/ml) of CerS1 using ELISA following 48 h treatment with cannabidiol at 10, 20 and 40 µM. (**e**) Western blot (s) of housekeeping gene, alpha-tubulin, CerS1, GRP78 and CHOP following siRNA treatment. (**f**) Western blot (s) of GRP78, CHOP and alpha-tubulin following 48 h treatment with cannabidiol at 10, 20 and 40 µM

siRNA knockdown of CerS1 exhibited a significant 94% knockdown in mRNA and 75% at 48 h in Panc03.27 cells (Fig. 4a and b). Western blot (Fig. 4e) for validating knockdown of CerS1 was difficult to observe as available antibodies didn’t work well therefore an ELISA (Fig. 4c) was also used to confirm knockdown. Protein quantification on ELISA confirmed knockdown of 81%, 70% and 64% following 48, 72 and 96 h respectively (Fig. 4c). Treatment with CBD at 10µM signficantly upregulated CerS1 by 38%, 20µM signficantly upregulated CerS1 by 66.5%, however 40µM of CBD treatment signficantly reduced CerS1 by 99% compared to untreated cells, possibly indicating doses above 20µM are highly toxic to sensitive cells (Fig. 4d). In order to analyse ER stress markers, knockdown of CerS1 on western blot showed downregulation of ER stress markers, GRP78 and CHOP and upon CBD treatment these markers were upregulated, indicating CBD induces ER stress which maybe driven by CerS1 (Fig. 4f). Overall knockdown of CerS1 is time dependent, hence the observed increases on ELISA and western blot detection which aligns with it’s known biochemical nature of high protein turnover as reported in the literature (Sridevi et al. 2009).

**Fig. 5:**
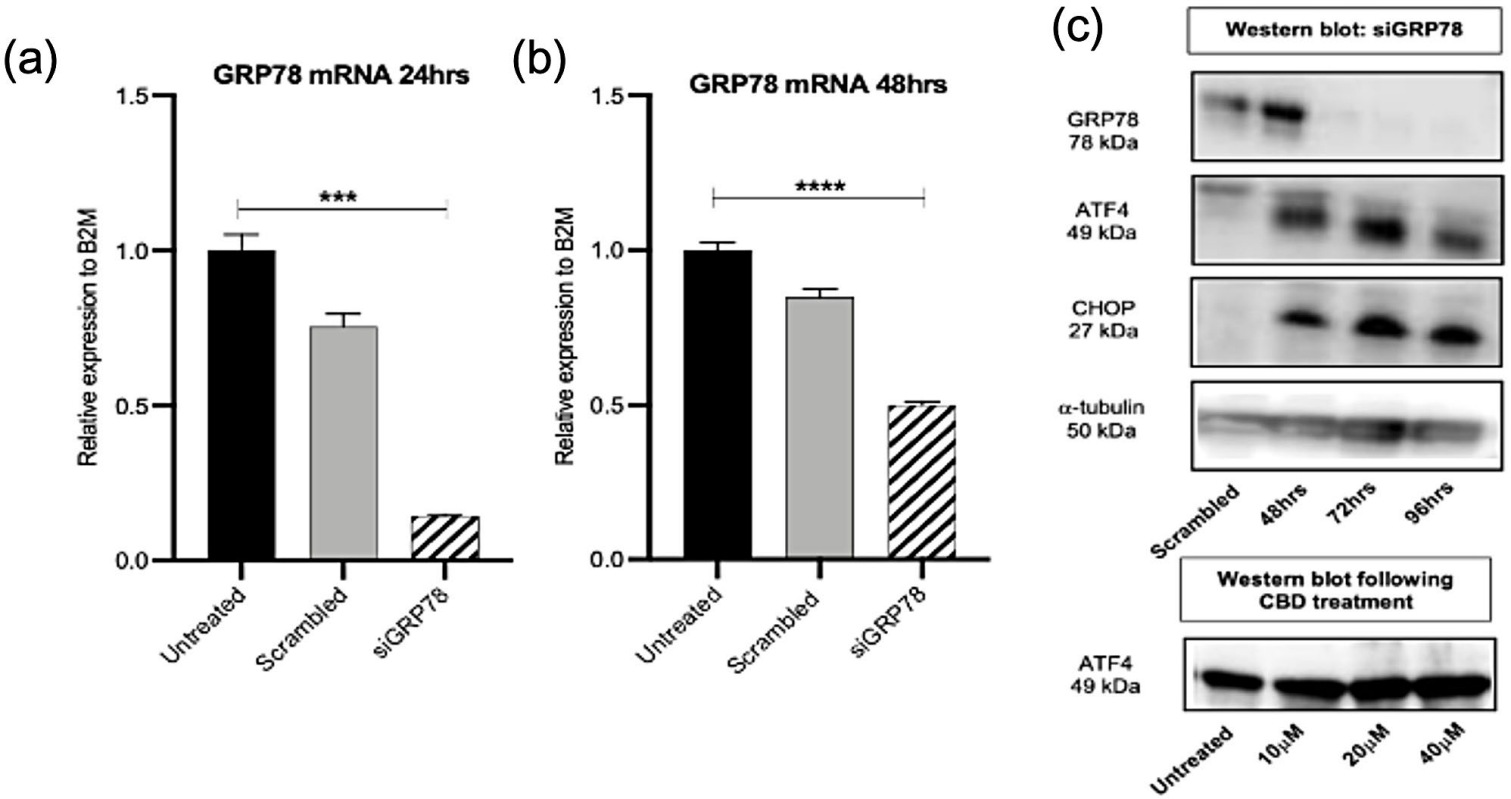
GRP78 knockdown in Panc03.27 cells does not affect the ATF/CHOP arm of the UPR pathway. (**a**) and (**b**) qPCR for GRP78 mRNA following transfection with 5 nM siGRP78 for 24 and 48 h. (**c**) Western blot (s) of housekeeping gene, alpha-tubulin, GRP78, ATF4 and CHOP following siRNA treatment and below plot represents ATF4 following 48 h treatment with cannabidiol at 10, 20 and 40 µM in Panc03.27 cells

In order to determine GRP78’s involvement, knockdown of GRP78 showed a significant decrease of 99% (24 h) and 51% (48 h) of expression level in Panc03.27 (Fig. 5a and b). Protein expression was significantly reduced by 72 h and 96 h (Fig. 5c). Levels of ATF4 and CHOP were also analysed following this knockdown, which showed increased levels when compared to control (scrambled sample) (Fig. 5c). CBD treatment at 10, 20 and 40µM didn’t show changes to the level of protein expression of ATF4 (Fig. 5c). Therefore, knockdown of GRP78 increased ATF4 and CHOP expression in these cells.

#### CBD induces endoplasmic reticulum stress via GRP78/ATF4/CHOP in Panc1 cells

**Fig. 6:**
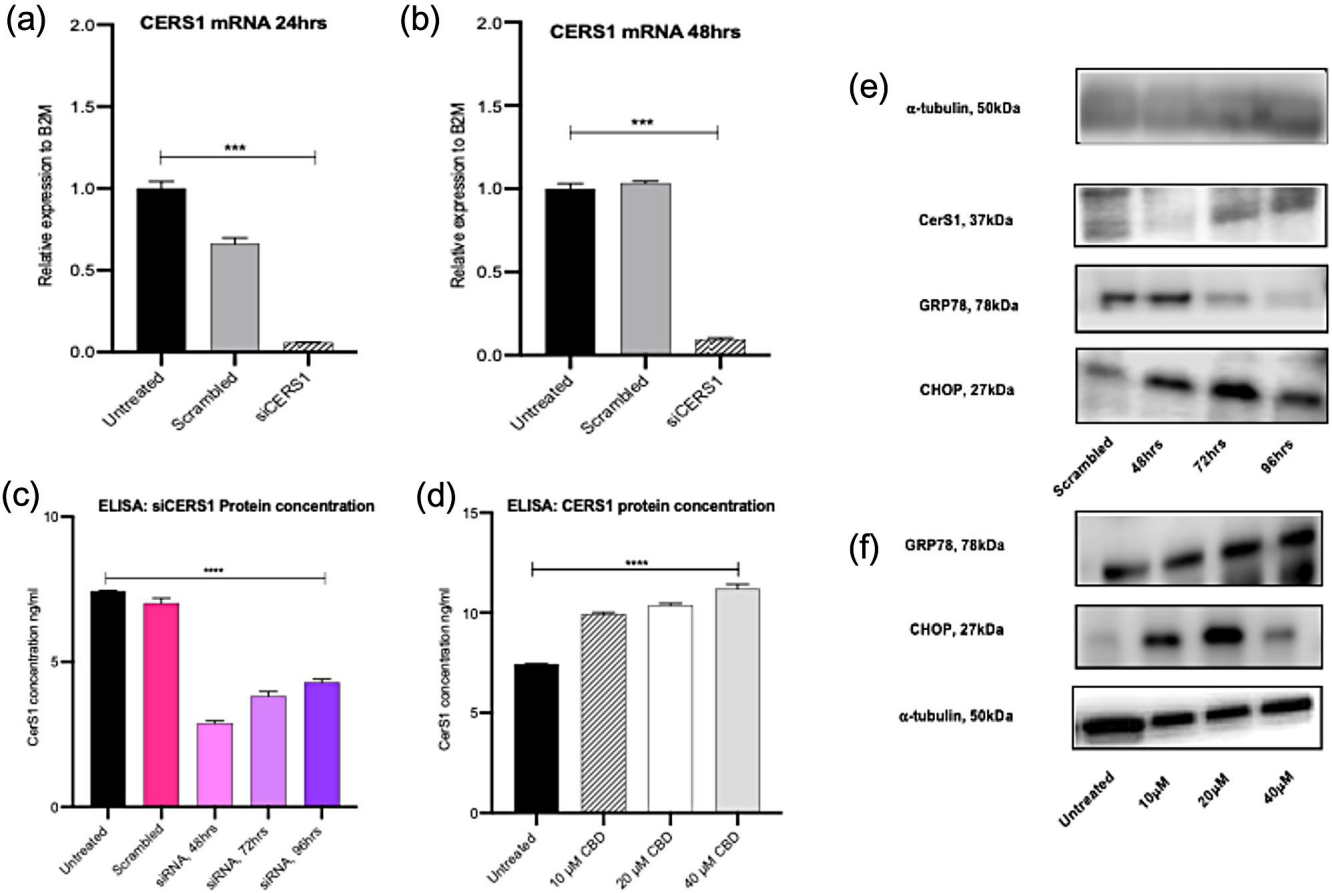
Ceramide synthase 1 knockdown causes reduction of GRP78 in Panc1 cells. (**a**) and (**b**) qPCR for CerS1 mRNA following transfection with 5 nM si-CERS1 for 24 and 48 h. (**c**) Bar blot of protein concentration (ng/ml) of CerS1 using ELISA following transfection with 5 nM si-CERS1 for 48, 72 and 96 h. (**d**) Bar blot of protein concentration (ng/ml) of CerS1 using ELISA following 48 h treatment with cannabidiol at 10, 20 and 40 µM. (**e**) Western blot (s) of housekeeping gene, alpha-tubulin, CerS1, GRP78 and CHOP following siRNA treatment. (**f**) Western blot (s) of GRP78, CHOP and alpha-tubulin following 48 h treatment with cannabidiol at 10, 20 and 40 µM

To validate and compare the findings observed in Panc03.27, similar investigations followed in gemcitabine resistant cells; Panc1 cells to determine CBD’s mechanistic actions. siRNA knockdown of CerS1 exhibited a significant 99% knockdown in mRNA at both 24 and 48 h in Panc1 cells (Fig. 6a and b). Protein expression on ELISA confirmed knockdown of 61%, 49% and 42% following 48, 72 and 96 h respectively (Fig. 6c). Treatment with CBD at 10µM signficantly upregulated CerS1 by 33.5%, 20µM signficantly upregulated CerS1 by 39.5%, 40µM of CBD treatment by 50.4% compared to untreated cells (Fig. 6d). In comparison to Panc03.27 cells, the knockdown of CerS1 showed downregulation of ER stress marker, GRP78 in comparison to CHOP and upon CBD treatment GRP78 and CHOP expression was greater when compared to untreated cells. However, 40µM of cannabidiol reduced CHOP expression in Panc1 cells which could be due to it’s resisistive quality these cells exhibit towards gemcitabine (Fig. 6e).

**Fig. 7:**
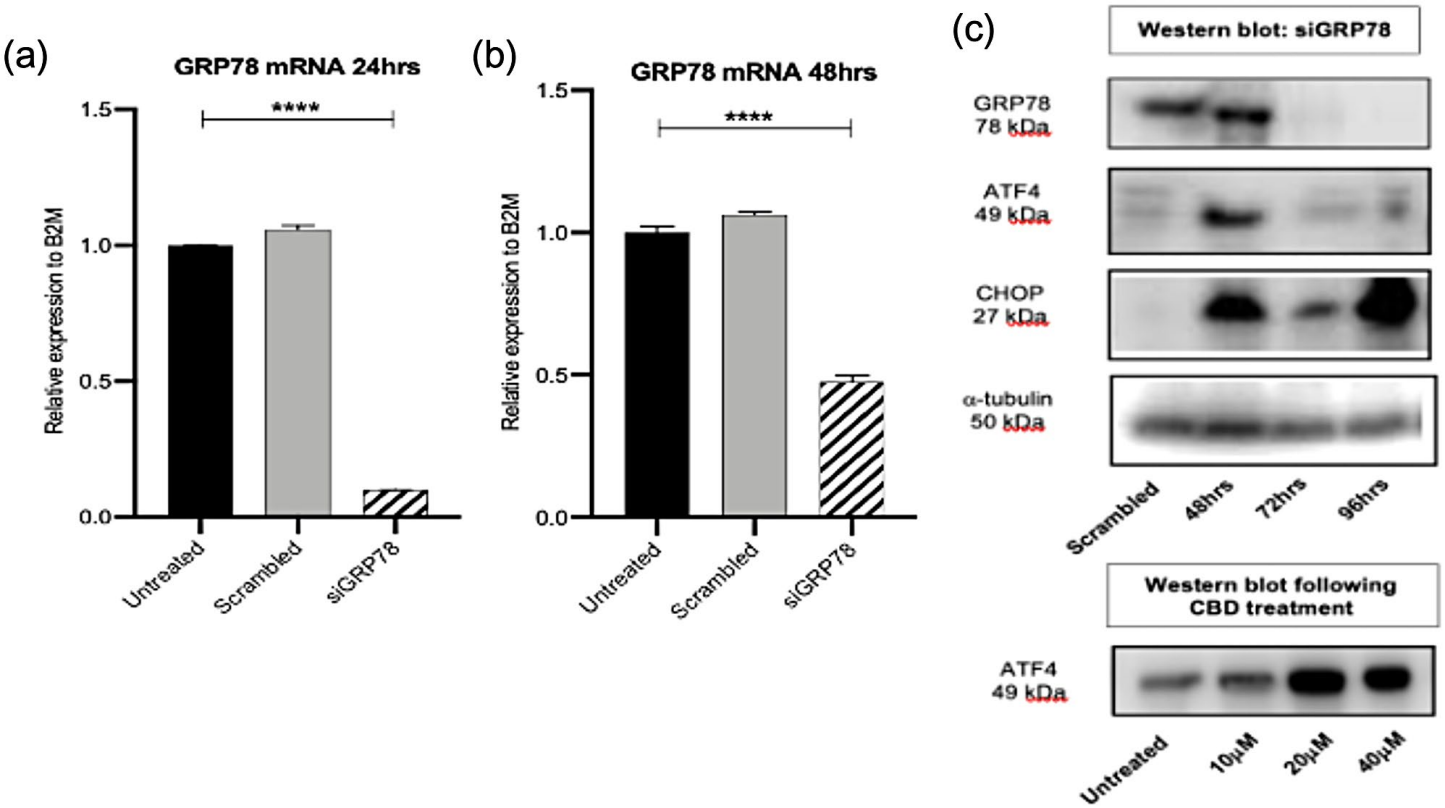
GRP78 knockdown in Panc03.27 cells does not affect the ATF/CHOP arm of the UPR pathway. (**a**) and (**b**) qPCR for GRP78 mRNA following transfection with 5 nM siGRP78 for 24 and 48 h. (**c**) Western blot (s) of housekeeping gene, alpha-tubulin, GRP78, ATF4 and CHOP following siRNA treatment and below plot represents ATF4 following 48 h treatment with cannabidiol at 10, 20 and 40 µM in Panc03.27 cells

In Panc1 cells, a significant 99% knockdown at 24 h and a significant 53% knockdown at 48 h in GRP78 mRNA expression was observed (Fig. 7a and b). Knockdown of GRP78 at protein level was determined by western blot and showed no expression following 48 h treatment with si-GRP78 (Fig. 7c). Interestingly, upon GRP78 silencing following 72 h, both ATF4 and CHOP protein expression levels were reduced which illustrates that in these chemo-resistant cells, silencing GRP78 decreases ATF4/CHOP arm of the UPR stream. Upon CBD treatment with 20µM and 40µM, ATF4 protein expression is enhanced compared to untreated cells (Fig. 7c).

We next investigated if CerS1 has a role in survival benefit in an animal model of PDAC. The aims of the study were firstly to analyse whether CBD could reduce tumour burden as both a monotherapy and or in combination with chemotherapy drugs gemcitabine and abraxane which are the standard care of treatment for patients with advanced PDAC. Secondly, to determine if there was an improvement in survival of these animals with CBD as an adjunctive to chemotherapy drugs. Thirdly, to determine CerS1 and GRP78 expression levels in excised tumours.

#### In an in vivo model where CerS1 is not upregulated, no survival benefit is seen from CBD monotherapy and gemcitabine combination

**Fig. 8:**
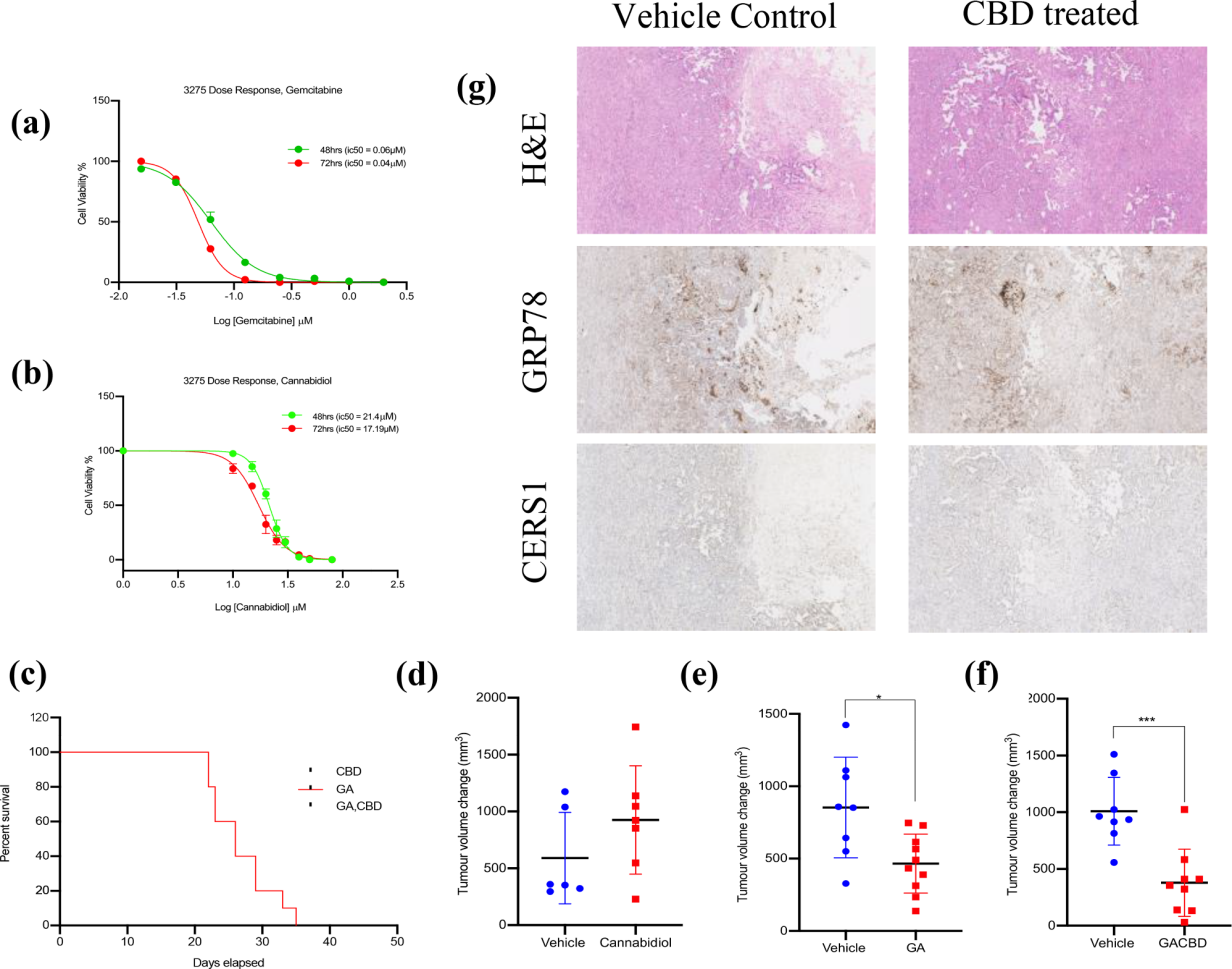
CerS1 upregulation may be needed to induce tumour reduction by cannabidiol (CBD) and increase survival in an in vivo model of PDAC. (**a**) Dose-response curve for gemcitabine treatment over 48 and 72 h. (**b**) Dose-response curve for cannabidiol treatment over 48 and 72 h. (**c**) Kaplan-meier curve for cannabidiol, gemcitabine and abraxane (GA) and gemcitabine, abraxane and cannabidiol (GACBD) groups. (**d**) Tumour volume change (mm^3^) for vehicle control group and cannabidiol treatment. (**e**) Tumour volume change (mm^3^) for vehicle control group and GA treatment. (**f**) Tumour volume change (mm^3^) for vehicle control group and GACBD treatment. (**g**) H&E and IHC staining for GRP78 and CerS1 in vehicle (control) and CBD treated tumours

Treatment of the murine cell line with gemcitabine and CBD showed a reduction in cell viability over 48 and 72 h (CBD; 48 h: 21.4µM and 72 h:17.1µM, Gemcitabine; 48 h:0.06µM and 72 h: 0.04µM) (Fig. 8a and b). In vivo, gemcitabine, abraxane and CBD triple combination treatment did not show a significant increase in survival however a reduction of 62.5% in tumour volume was observed which was significant (*p* = 0.0006) when compared to their respective vehicles (Fig. 8f). CerS1 was not upregulated in the tumour sections upon immunohistochemistry staining (Fig. 8g) which may indicate that CerS1 induced cytotoxicity is induced by CBD.

## Discussion

The work highlights the following: CBD has cytotoxic effects which are dose-dependent and time dependent in micromolar concentrations in both human and murine pancreatic cancer cell lines. In this study, the use of IMR-90 which are lung fibroblasts and H6c7 (HPDE) immortalised epithelial cells from normal human pancreatic ductal cells showed cytotoxicity at higher micromolar concentrations with CBD treatment (55µM, 50µM (48 h) and 51µM, 47µM (72 h) respectively) when compared to the cancer cells, indicating that pancreatic cancer cells were more sensitive to CBD treatment compared to IMR-90 and H6c7 (HPDE). Secondly, the cytotoxic mechanism of action of CBD is CerS1 dependent driven which induces endoplasmic reticulum stress leading to unfolded protein response via an activation of ATF4 and CHOP. The findings show CBD upregulates CerS1 in both gemcitabine sensitive (Panc03.27) and resistant (Panc1) cells. The addition of gemcitabine enhances the upregulation of CerS1 in Panc03.27 cells compared to Panc1 cells. An increase in CerS1 initiates GRP78 and downstream ATF4 and CHOP pathway, and protein expression shows a profound involvement of ATF4/CHOP on knockdown of GRP78 in Panc1 cells. In sensitive cells the combination of gemcitabine and CBD produced a greater effect of ceramide inducing ER stress in comparison to resistant cells. CBD as a single treatment could induce ER stress independently of gemcitabine in resistant cells, Panc1.

Cannabinoids are unlikely to be used clinically as monotherapies in cancer treatment, and there is initial evidence that they could serve as cytotoxic adjuvants with chemotherapy (Ferro et al. 2018; Griffiths et al. 2021; Mangal et al. 2021). The aim of the in vivo study was to establish whether CBD could sensitise a cell line derived xenograft mouse model of PDAC to gemcitabine and abraxane. The findings of our in vivo model of CBD treatment did not show survival benefit in the animals which may be indicative of the fact that CerS1 was not upregulated in the treated tumours with CBD (Fig. 8g), although in combination with chemotherapy drugs; gemcitabine and abraxane a significant tumour burden reduction was observed. These results warrant further investigation of the dosing concentration required in vivo to elicit the response seen in vitro.

Many animal studies have shown varying outcomes when examining CBD’s oncological effects in vivo. CBD is highly lipophilic and can precipitate in the gastro-intestinal (GI) tract which results in a slower absorption than elimination rate, CBD is also highly sensitive to light, temperature, and oxidation (Millar et al., 2020). Studies have reported various dosing of CBD ranging from 5 mg/kg to 100 mg/kg in murine models of cancer which emphasises the complexity of a “dosing” which would be effective across all models. In the study by Ferro et al., they report the use of 100 mg/kg of CBD and gemcitabine at 100 mg/kg, whereas in our study we used 10 mg/kg of gemcitabine indicating that these differences could also factor into the antitumour effects they observe (Ferro et al. 2018; Mangal et al. 2021). Additionally, drug-drug interactions are a problem with CBD intake as it involves metabolism by a competitive inhibitor of CYP450 enzymes which are essential enzymes involved in production of cholesterol, steroids, prostacyclin’s, and thromboxane A2, which could cause drug metabolite levels to be altered (Brown and Winterstein 2019; Lynch and Price 2007).

Therefore, methods to increase CBD bioavailability such as using self-emulsifying drug delivery systems, targeted delivery such as the use of a cancer specific RNA-aptamer and improved formulation of the drug should all be considered for a future “effective” therapy formulation. Finally, using a 3D model such as organoids which recapitulates the tumour microenvironment, would be an ideal platform to use to study the antitumour effects of CBD in order to supplement the in vivo data.

Overall, this study points to a potential CerS1 dependent driven pathway activated by CBD treatment which leads to downstream GRP78, ATF4 and CHOP activation causing cellular stress and cell death. It is interesting to note that CBD behaves differently in cells which are sensitive or resistant to chemotherapy. This warrants further investigation to compare against additional sensitive versus resistant lines to determine cell specific effects of CBD in its cytotoxic mechanistic action.

## Conclusions

The findings presented in this work, indicate dose-dependent and time-dependent cytotoxic effects of CBD in both human and murine pancreatic cancer cells. Gemcitabine and CBD in combination upregulate CerS1 greater in gemcitabine sensitive cells and in resistant cells CBD alone can upregulate CerS1. The downstream effects of CerS1 upregulation induce ER stress which activate the GRP78/ATF4/CHOP arm of the UPR response in gemcitabine resistant cells. Our pilot in vivo study showed a significant reduction in tumour volume in both double chemotherapy and combination of all three; CBD, gemcitabine and abraxane. No significant difference in survival was observed for CBD alone and as an adjunct therapy. The in vitro platform points to a potential novel mechanism of CBD’s mechanism of action in pancreatic cancer cells and further animal studies are needed to validate ceramide’s involvement in CBD therapy for PDAC.

### Experimental procedures

#### Cell lines, culture media and conditions

Panc03.27, Panc1, HPAF-II, CFPAC-I, IMR-90 were cultured according to ATCC’s guidelines. and murine-3275 were cultured in RPMI supplemented with 10% FBS. Panc03.27 cells were seeded in respective 96 well plates at 1 × 10^4^ and Panc1 at 8 × 10^3^ for the use of CellTiter-Glo® (Promega, Cat No G9241) and Sulforhodamine B (SRB) (Abcam Cat No ab235935) assays to analyse cytotoxicity of drug treatments.

### Transfections

Cell lines Panc03.27 and Panc1 were seeded in a 24-well plate at 7.5 × 10 and 5 × 10 respectively and treated with following siRNA targets, Silencer™ Select Negative Control No. 1 siRNA (cat no.: 4390843), Silencer® Select Pre-Designed & Validated siRNA s6980 (cat no.: 4392420), Silencer® Select Pre-Designed & Validated siRNA s230924 (cat no.: 4390816) and DsiRNA DDIT3, Integrated DNA Technologies (cat no.: hs. Ri. DDIT3.13.1). Transfection was performed after 4–6 h of seeding (until cells adhered) with the indicated oligonucleotide concentration using 1µL per well of Lipofectamine RNAiMAX according to manufacturer’s instructions (ThermoFisher, Invitrogen, Protocol Pub. No. MAN0007825 Rev.1.0). Transfections were conducted with Lipofectamine RNAiMAX (ThermoFisher, Cat No 13,778,150). Replacement of medium took place 24 h later containing drugs for treatment. At the 24- and 48-hours’ time point post treatment, cells were harvested for analysis. Silencer™ Select Negative/Scrambled Control No.1 siRNA, catalgoue number; 4,390,843, Silencer® Select Pre-designed & validated siRNA s6980 (BiP/GRP78), catalogue number; 4,392,420, Silencer® Select Pre-designed & validated siRNA s230924 (CERS1), catalogue number; 4,390,816.

### RNA extraction and real-time PCR

Total RNA was isolated from cultured cells using the RNeasy Mini Kit according to manufacturer’s instructions (QIAGEN). The RNA was quantitated using a QIAxpert microfluidic spectrophotometer (QIAGEN) and reverse transcribed using the Quantitect Reverse Transcription Kit according to manufacturer’s instructions (QIAGEN). Relative mRNA expression levels were determined by real-time PCR using either Applied BiosystemsTM PowerUpTM SYBRTM Green Master Mix (Applied Biosystems) with validated QuantiTect SYBR Probes from QIAGEN or TaqMan Fast Advanced Master Mix (Life Technologies) with validated FAM Probes from Applied Biosystems. Following Quantitect Primer Assays (QIAGEN) were used for data analysis to ensure accurate interpretation of the data and in line with the MIQE guidelines: Hs_B2M_1_SG (QT00088935), Ms_B2m_1_SG (QT01149547), Mm_Cers1_1_SG (QT00151081), Mm_Cers2_1_SG (QT00144025), Mm_Cers3_1_SG (QT00525952), Mm_Cers4_1_SG (QT00122101), Mm_Cers5_1_SG (QT00101605), Mm_Cers6_1_SG (QT00137291). The following FAM Probes purchased from Applied Biosystems that were used are listed: B2M (Hs00187842_m1), AHSA1 (Hs00201602_m1), ATF4 (Hs00909569), GRP78 (Hs00607129), CERS1 (Hs04195319_s1), CERS2 (Hs00371958_g1), CERS3 (Hs00698859_m1), CERS4 (Hs00226114_m1), CERS5 (Hs00332291_m1), CERS6 (Hs00826756_m1). Relative gene expression values were calculated using the Livak method 2^-(ΔΔCt), where target genes were normalised to a housekeeping gene (Livak and Schmittgen 2001).

### Western blot

Cells were seeded at different seeding densities per well in a 24-well plate depending on each cell line and transfected as described above. A pool of 4 wells per condition was used for total protein extraction. 48, 72 and 96 h post drug/siRNA treatment and prior to cell lysis, all wells were rinsed twice with either ice cold Phosphate Buffered Saline (PBS) or Tris Buffered Saline (TBS) buffer in the case of phosphoproteins only. RIPA lysis buffer was then added to cells (100µL/well) containing 50mM of Tris, 150 mM NaCl, 0.1% SDS, 0.5% sodium deoxycholate, 1% NP40 and 5mM EDTA supplemented with protease inhibitor cocktail (Sigma, Cat No P8340) and phosphatase inhibitor cocktail (Sigma, Cat No 524,629). Thereafter, the cells were transferred into pre-chilled tubes using a cell scraper and centrifuged gently for 15 min at 5 °C. Cell debris was subsequently removed, and the protein supernatant was transferred into a pre-chilled tube. The amount of protein per condition was thereafter quantified using the RC-DC Bradford Assay Kit following the manufacturer’s protocol (Bio-Rad, Cat No 5,000,120), and 20 µg of total protein was then loaded onto SDS-PAGE (ThermoFisher, Cat No XP04122BOX). A recombinant protein of human CERS1 (ORIGENE, Cat No TP311201) was also purchased and loaded as a positive control alongside with the CERS1 samples for the validation of CERS1 protein levels. The acrylamide gels were then transferred onto polyvinylidene fluoride (PVDF) (Sigma, Cat No GE10600023) membranes for western blotting using the antibodies; Anti-ATF4 (Cell signalling, 11,815), Anti-Ceramide Synthase 1 (Abcam, ab85696), Anti-alpha tubulin (Abcam, ab7291), Anti-GRP78 (Cell Signalling, 3177), WesternSure® HRP Goat anti-Mouse IgG (H + L) (LI-COR, 926-80010), WesternSure® HRP Goat anti-Rabbit IgG (H + L) (LI-COR, 926-80011), Donkey Anti-Goat IgG H&L (HRP) (Abcam, ab205723).

### ELISA

Following cell lysis (see 4.5 western blot), 100µL of sample, standards, blanks were added to the pre-coated microtiter plate wells with a biotin-conjugated antibody preparation specific for target antigen and then avidin conjugated to Horseradish Peroxidase (HRP) was added to each microplate well and incubated followed by the addition of a TMB substrate solution added to each well. Only those wells that contain target antigen, biotin-conjugated antibody and enzyme-conjugated Avidin will exhibit a change in colour. The enzyme-substrate reaction was terminated by the addition of a sulphuric acid solution and the colour change measured spectrophotometrically at a wavelength of 450 nm +/- 2 nm. The concentration of target antigen in the samples was then determined by comparing the O.D. of the samples to the standard curve, as referred to in MyBioSource, Human CERS1 ELISA kit (Cat no. MBS2890964).

### Mice, tumour induction and cell isolation

3275-Luc^+^ cell line was derived from a KPC model on a C57/BL6 background harbouring mutation in KRAS, INK4A and p53 (derived from the *Swiss* Institute for Experimental Cancer Research (ISREC)). Tumours were excised, meshed, and processed to form the cell line. 3275-Luc^+^ cells were cultured in RPMI 1640 (Lonza, Cologne, Germany) supplemented with 10% FCS for injection preparations.

All in vivo experiments were performed in accordance with the local ethical review panel, the UK Home Office Animals (Scientific Procedures) Act 1986, the United Kingdom National Cancer Research Institute guidelines for the welfare of animals in cancer research, and the ARRIVE guidelines. 70 FVB/NJ female mice aged between 6 and 8 weeks old were purchased from Charles Rivers, Germany at a mean weight of 23 g and used in this study. 3275-Luc^+^ cells were screened for mycoplasma. Animals were housed in specific pathogen-free rooms in autoclaved, aseptic micro isolator cages with a maximum of five animals per cage. For tumour induction, 0.5 × 10^6^ 3275-Luc^+^ cells in 100uL volume of HBSS were injected subcutaneously in the right flank of FVB/N mice post-acclimatization. Animals were checked twice weekly for good health, ulceration grade and tumour growth. However, any mice displaying the following pre-assigned effects were culled. The pre-assigned end points included mice displaying one of the following: development of abdominal ascites, severe cachexia, significant weight loss (approaching 20% of initial weight), extreme weakness, inactivity, discomfort, or pain. No major side/adverse effects and no weight loss were observed in mice treated with CBD. Tumour measurements were performed using a caliper with width and height noted and volume calculated using the formula: 4.19*(d/4 + d/4)^3^. Once tumour reached 5 mm x 5 mm in size, mice were randomised to treatment/control groups based on mean tumour volumes using a record table on excel software. Mice were then treated according to their appropriate arm of treatment. Endpoint for each animal was determined by tumour burden or G4 ulceration. Mice were sacrificed and tumours were removed for cell isolation in MACS® Tissue Storage Solution (Miltenyi Biotec). Tumours were spun at 200xg for 5 min, then meshed and passed through a 100 μm cell strainer (Miltenyi SmartStrainer) to obtain a single cell suspension using collagenase and DNase (Sigma-Aldrich, Munich, Germany) and incubated at 37 °C for 40 min. Tumour cells were filtered through a 100 μm mesh, washed, and stained for flow cytometer analysis.

### Drug and vehicle injections

Drug treatments were administered via intraperitoneal means and for the triple combination arm, injection sites were altered from right to left flank. Gemcitabine (purchased from MedChemExpress, United Kingdom) and vehicle (DMSO) were injected at 10 mg/kg twice weekly, Abraxane (nab-paclitaxel purchased from Celgene, Netherlands) and vehicle Albumin human (purchased from Sigma Life Science) were injected at 10 mg/kg twice weekly and Cannabidiol and vehicle (DMSO) were injected at 100 mg/kg thrice weekly. Cannabidiol, 100% purity, was obtained from EMMAC life sciences (Batch no. MCE/CBD/19 − 001) and dissolved in Tween-80 (Sigma-Aldrich), sunflower oil (Sigma Life Science) and PBS (Gibco) at a 1:1:8 ratio in 0.01% DMSO.

### Immunohistochemistry on formalin fixed and paraffin embedded tissue

3 μm sections of formalin-fixed, paraffin-embedded (FFPE) tissues were cut using the Leica RM2255 microtome (Leica Biosystems Ltd., Newcastle). Prior to immunostaining, sections were deparaffinised in xylene, re-hydrated through a series of decreasing concentrations of ethanol and transferred to water. In detail, slides were immersed in xylene solution for x2 10 min following by re-hydration progressively in ethanol 100% for 5 min, ethanol 96% for 5 min, ethanol 80% for 5 min and H_2_O for 5 min. Sodium citrate buffer [10mM; 0.05%Tween-20 (pH = 6.0)] was used for antigen retrieval process and slides were placed in glass jars and boiled in water bath for 30 min at 110 °C. Sections were left to cool down gradually at room temperature. Slides were washed by Tris-buffered saline (TBS) − 0.025% Tween 86 twice for 5 min each, incubated in a dark humidified chamber at room temperature with 5% BSA (400µL of BSA (12.5%) and 600µL of PBS-Triton 0.25%). Immediately, without washing, primary antibody in PBS + TritonX100 + 1% BSA added and incubated overnight at 4˚C temperature. Slides were washed three times for 5 min each in TBS-0.025% Tween 20 and secondary antibodies added and left at room temperature for 60 min. Following by washing with TBS-0.025% three times for 5 min each in order to remove excess of secondary polymer antibody. Signal detection was done using diaminobenzidine tetrahydrochloride (DAB) as the reaction of chromogenic for 5 min. Reaction stopped by immersing the slides with ddH2O and then counterstained with Haematoxylin (50% Harris-50%Mayer) (VWR International Ltd., Leicestershire, UK) for 5 s and briefly washed in tap water. Dehydration applied with 3 min incubation with 96% ethanol, 10 min with 100% ethanol and 5 min in xylene. Slides were sealed with a drop of mounting reagent (VWR International Ltd., Leicestershire, UK) and coverslips (VWR International Ltd., Leicestershire, UK).

### Statistical analysis

Students two-tailed t test was used to compare vehicle and drug/siRNA treated groups. A p-value of less than 0.05 was considered significant. As shown on the graphs, * denotes *p* < 0.05, ** denotes *p* < 0.01 and *** denotes *p* < 0.001 in comparison to vehicle. All graphs were generated with GraphPad Prism 8 (GraphPad Software, USA).

Kaplan-Meier estimator used to estimate the proportion of mice alive at any one time-point in the study groups. It is a nonparametric test and appropriate to use when the data are right skewed and censored.

## Data Availability

Not Applicable.
